# Behavioral variant frontotemporal dementia associated with *GRN* and *ErbB4* gene mutations: a case report and literature review

**DOI:** 10.1186/s12920-024-01819-5

**Published:** 2024-01-30

**Authors:** Youde Cai, Zhongyong Peng, Qiansong He, Ping Sun

**Affiliations:** 1 Department of Neurology, The Second People’s Hospital of Guiyang, No. 547 Jinyang South Road, Guiyang, Guizhou Province 550000 China; 2 Department of Neurology, The First Affiliated Hospital of Guizhou University of Traditional Chinese Medicine, Guiyang, Guizhou Province 550000 China

**Keywords:** Behavioral variant frontotemporal dementia, *GRN* and *ERBB4* genes, Mutation, Abnormal behavior, Unstable walking

## Abstract

**Objective:**

To report the clinical manifestation and genetic characteristics of a patient having frontotemporal dementia (FTD) with abnormal behavior and unstable walking.

**Methods:**

The clinical and imaging features of a patient who was eventually diagnosed with FTD were analyzed. The patient’s neuropsychological, PET-CT, electromyography, and genetic data were collected. Furthermore, the patient’s blood samples were examined for FTD-related genes.

**Results:**

The patient was a 52-year-old man with hidden onset. The symptoms progressed gradually, presenting with abnormal behaviors, including repeated shopping, taking away other people’s things, constantly eating snacks, and frequently calling friends at night. The patient also exhibited executive dysfunction, such as the inability to cook and multiple driving problems, e.g., constantly deviates from his lane while driving. In addition, the patient showed personality changes such as irritability, indifference, and withdrawal, as well as motor symptoms, including unstable walking and frequent falls when walking. Brain magnetic resonance imaging revealed hippocampal sclerosis along with widening and deepening of the bilateral temporal lobe sulcus. Brain metabolic imaging via PET-CT demonstrated decreased metabolism in the bilateral prefrontal lobe, with the abnormal energy metabolism indicating FTD. Lastly, blood sample analysis detected mutations in the amyotrophic lateral sclerosis (ALS)-related *GRN* gene c.1352C > T (p.P451L) and *ErbB4* gene c.256 T > C (p.Y86H).

**Conclusion:**

This is the first case of heterozygous mutations in the *GRN* and *ErbB4* genes in FTD alone. The *GRN* and *ErbB4* genes are likely to be important in the pathogenesis of FTD, expanding the common genetic profile of ALS and FTD.

## Introduction

Frontotemporal dementia (FTD) is a neurodegenerative disease that progressively destroys the nerve cells in the frontal and temporal lobes of the brain [1] and is the second most common form of dementia in individuals before the age of 65 years [2]. Clinically, the disease is characterized by behavioral changes, impaired frontal lobe executive function, and/or language disorders [3], accounting for 5–10% of all dementia types and approximately 10–20% of cases in patients < 65 years [4]. Familial FTD is observed in 20–50% of patients with FTD, with 10% exhibiting a significant autosomal dominant genetic pattern [5, 6]. FTD is a group of clinical syndromes, mainly including semantic variant primary progressive aphasia (svPPA), nonfluent variant of primary progressive aphasia (nfvPPA), and behavioral variant frontotemporal dementia (bvFTD). The most common FTD syndrome is bvFTD [7], a clinical syndrome characterized by early changes in personality and behavior. As FTD progresses, the personality, social behavior, and cognition of the patients gradually deteriorate [8]. Previous studies have shown that FTD and amyotrophic lateral sclerosis (ALS) are neurodegenerative diseases, with up to 22% of patients with ALS meeting the diagnostic criteria for FTD [9]. Moreover, about 15% of patients with FTD present with signs of motor neuron disease, and both FTD and ALS have an autosomal dominant pattern in 10% of cases. Consequently, they may share common pathophysiological features. In this study, the mutation of the ALS pathogenesis-associated progranulin (*GRN*) gene c.1352C > T (p.P451L) and the *ErbB4* gene has a heterozygous mutation c.256 T > C(p.Y86H) was found in our patient during the analysis of FTD-related genes. Therefore, our study investigates the role of *GRN* and *ErbB4* genes in the pathogenesis of FTD and provides further theoretical support that ALS and FTD extend the common genetic profile of ALS and FTD.

## Case presentation

### Clinical information

Our patient was a 52-year-old right-handed man with 5 years of primary school education. The patient was admitted to The Second People’s Hospital of Guiyang City in Guizhou Province on November 14, 2020, because of personality changes with unstable walking for > 2 months. The patient’s family reported that he had exhibited abnormal behavior for 2 months, mainly manifesting as repeated online shopping, placing other people’s things in his bags, constantly calling his friends at night, cursing when greeted by younger family members, inclination toward foul language, not paying attention to hygiene, and constantly eating snacks. The patient also showed personality changes such as irritability, indifference, indifference to family and friends, and impatience. Furthermore, the patient exhibited many driving issues, e.g., always deviating from his lane and not using the brakes when driving downhill, as well as other executive dysfunction, including cooking without seasoning and slow response during card games. The patient also experienced poor sleep quality and memory disorders such as forgetting the content and date of a conversation, repeatedly asking or discussing the same questions, inability to place items back in the original position, and abandoning things. Additionally, the patient demonstrated motor symptoms, characterized by unstable gait, such as limb weakness, slight walking instability and experienced frequent falls while walking. Upon detailed examination, the gait instability was noted to be non-specific and did not show classic signs of pyramidal tract involvement, parkinsonism, or frontal gait apraxia. Moreover, the patient had no obvious abnormality in the stool and urine samples and no significant change in body weight. In the case of medical, personal, and family histories, the patient had a history of smoking for > 30 years (an average of 5 cigarettes per day) but no history of drinking. The family history showed no cognitive impairment, mental illness, or reported consanguineous marriages.

Physical examination on admission indicated a body temperature of 36.6 °C, pulse rate of 77 beats/min, respiratory rate of 19 breaths/min, and blood pressure of 132/88 mmHg. No abnormal findings were detected during the heart, lung, and abdominal examinations. Neurological examination revealed a clear and active consciousness, answers are not relevant, and Memory loss. Ophthalmological investigation revealed normal eye movement with no nystagmus. However, additional ophthalmological tests indicated bilateral pupil sizes were 2 mm:3 mm (left eye:right eye), with the left pupil showing photosensitivity and the right presenting with light insensitivity. Further examination of the patient revealed symmetrical bilateral forehead lines and that the frown lines were not crooked. The facial needling sensation was also symmetrical, and the tongue was in the center. Muscle function tests demonstrated that the extremities had a 5-grade muscle strength and moderate muscle tension, and the patient had no muscle atrophy. Moreover, the tendon reflex was symmetrical, with no detection of any pathological signs. In addition, he had negative meningeal stimulation sign. However, the patient had an unstable walk and could not walk in a straight line, whereas the rest of the coordination function was stable.

A mental examination of the patient revealed that he would enter the ward by himself, as well as displayed problems involving expressions at ease, delayed thinking, slow to respond, persecutory delusion, slightly anxious mood, and worry. The following scores were obtained on neuropsychological assessment: a score of 4.5 on the Frontotemporal Lobar Degeneration (FTLD)-modified Clinical Dementia Rating (CDR), 47 on the Frontal Behavioral Inventory (FBI) (where FBI-A score ≥ 30 indicates FTD), 63 on the Mild Behavioral Impairment Checklist (MBI-C), 23/30 on the Mini-Mental State Examination (MMSE), 19/30 on the Montreal Cognitive Assessment (MoCA), 78 on the Neuro-Psychiatric Inventory (NPI) patient, 23 on the NPI caregiver, 11 on the Geriatric Depression Scale (GDS), and 22 on the Activities of Daily Living (ADL). Table 1 displays the scores of the neuropsychological scales.

**Table 1:** Neuropsychological scale scores of the patient

Scales	Scale scores
MMSE	23
MoCA	19
CDR(Summation)	2.5
FTLD_CDR	4.5
AVLT_Immediate memory	13
AVLT_Delayed memory	4
AVLT_Cue memory	4
AVLT_Delayed recognition	10
Forward digit span	8
Reverse digit span	5
A connection time (seconds)	112
A connection correct line	25
B connection time (seconds)	102
B connection correct line	20
Boston Naming Test	17
NPI_Patient	78
NPI_Caregiver	23
GDS	11
ADL	22

*MMSE* Mini-Mental State Examination, *MoCA* Montreal Cognitive Assessment, *ADL* Activity of Daily Living, *FTLD-CDR* Frontotemporal Lobar Degeneration Clinical Dementia Rating Scale, *AVLT* auditory verbal learning test, *NPI* Neuro-Psychiatric Inventory, *GDS* geriatric depression scale, *ADL* a ctivities of daily living.

Auxiliary examination involving routine blood testing demonstrated the following findings: lymphocytes, 12.9%; eosinophils, 0.2%; neutrophils, 81.8%; lymphocyte count, 1.09 × 10^9^/L; neutrophil count, 6.95 × 10^9^/L; total cholesterol (blood lipids), 5.91 mmol/L; and homocysteine, 17.5 mmol/ L. Additionally, no obvious abnormality was detected in the routine blood tests for blood coagulation, folic acid, vitamin B12, liver and kidney function, electrolyte, infectious marker (HIV + TPab), thyroid function, urine, and stool + occult blood.

Lumbar puncture for cerebrospinal fluid analysis revealed a brain pressure of 140 mmH_2_O, and there were no abnormalities observed in the routine, biochemical, or cytological analysis of the cerebrospinal fluid. The electroencephalogram (EEG) results were within the normal range.

**Fig. 1:**
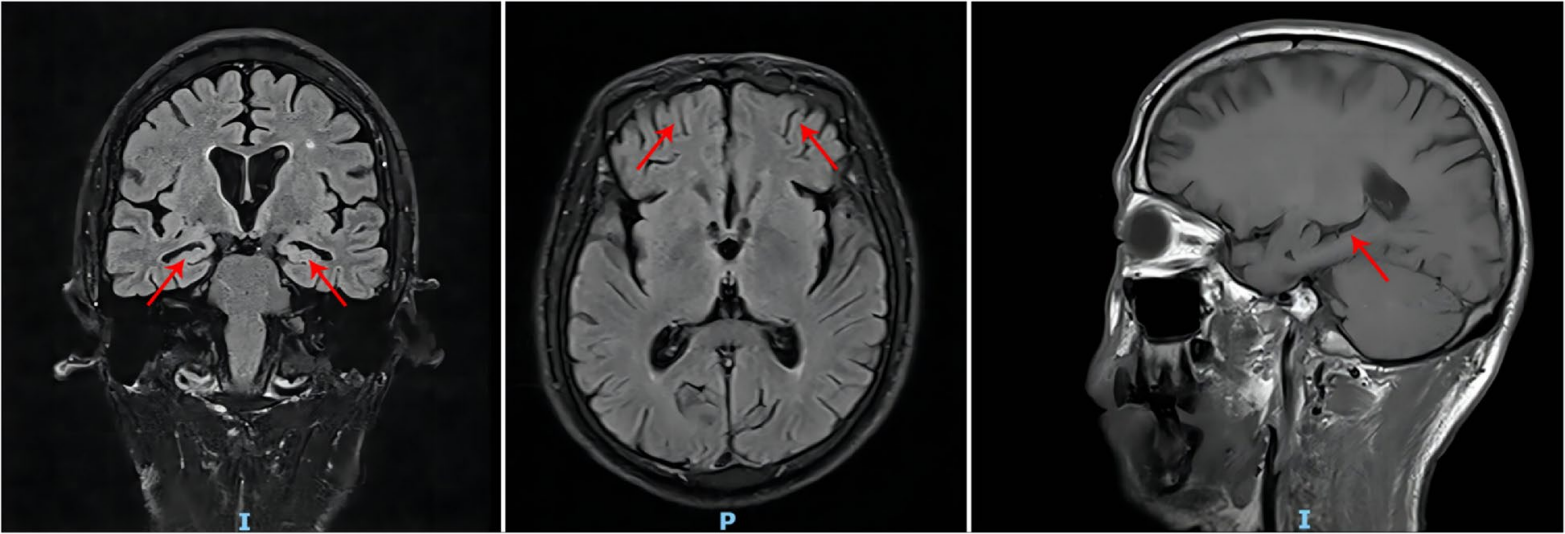
Brain imaging examination showing the brain abnormalities of the patient. Bilateral hippocampal atrophy, frontotemporal lobe atrophy, and widening and deepening of the bilateral temporal lobe sulcus were observed

**Fig. 2:**
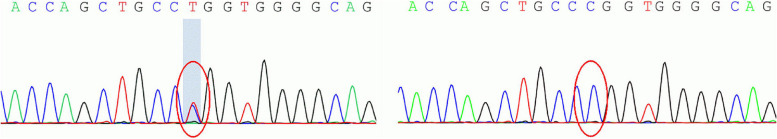
Sequence diagram of the *GRN* gene mutation in the patient and his daughter. High-throughput gene testing revealed a c.1352C > T (p.P451L) heterozygous mutation in the *GRN* gene while no variants were detected in his daughter

Electromyography (EMG) showed slight damage to the ulnar nerve of the right elbow. Brain magnetic resonance imaging (MRI; Fig. 1) indicated abnormal signals in the deep white and paraventricular matter, with a grade II on the Fazekas classification. In addition, multiple lacunar infarcts along with widening and deepening of the bilateral temporal lobe sulcus were observed. Furthermore, slightly decreased volume and increased signal in the bilateral hippocampus suggested hippocampal sclerosis. MR angiography demonstrated that the A1 segment of the right anterior cerebral artery was narrow and superficially visible. Brain metabolic imaging via positron emission tomography–computed tomography (PET-CT) showed decreased PDG metabolism in the bilateral prefrontal lobe, with abnormal energy metabolism implying FTD (Fig. 2).

Based on the patient’s clinical findings, neuropsychological assessments and neuroimaging results along with his patient’s behavioral symptoms and cognitive profiles, the patient was diagnosed as having bvFTD according to the “International Consensus Standard” [8] developed by Rascovsky K et al. in 2011.

### Detection method for FTLD gene mutations

After obtaining informed consent from the patient and his family, venous blood samples (5 ml) of the patient and his daughter were collected and sent to Beijing Jinzhun Gene Technology Co., Ltd. for high-throughput gene testing. The investigated genes included leucine-rich repeat kinase 2 (*LRRK2*),alpha-synuclein (*SNCA*), Microtubule-associated protein tau (*MAPT*), fused in sarcoma gene (*FUS*), coiled-coil-helix-coil-helix domain-containing protein 2 (*CHCHD2*), *GRN* and other cognitive and motor disorder genes. Briefly, genomic DNA was extracted from the blood samples using ethylenediamine tetraacetic acid as an anticoagulant. The DNA was then fragmented and used to construct a genomic library. Next, the DNA in the coding and adjacent splicing regions of the target gene was captured and enriched by the chip. Finally, the high-throughput sequencing platform was used for mutation detection.

### Genetic testing results for FTLD gene mutations

**Fig. 3:**
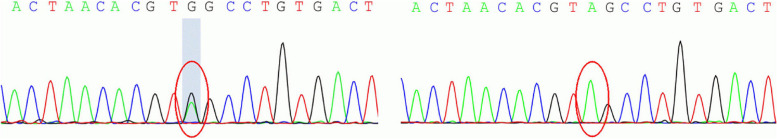
Sequence diagram of the *ErbB4* gene mutation in the patient and his daughter. The patient had a heterozygous mutation c.256 T > C (p.Y86H) in the ERBB4 gene while no variants were found in her daughter

The high-throughput gene testing revealed a c.1352C > T (p.P451L) heterozygous mutation in the *GRN* gene (nucleic acid 1352 in the coding region changed from C to T), leading to the conversion of amino acid 451 from proline to leucine (p.P451L). Similarly, a c.256 T > C (p.Y86H) heterozygous mutation was detected in the *ErbB4* gene (nucleic acid 256 in the coding region changed from T to C), resulting in the alteration of amino acid 86 from tyrosine to histidine (p.Tyr86His, Y86H), which was a missense mutation. Consequently, the mutation of *GRN* and *ErbB4* may cause damage to the structure and function of the corresponding protein, but its pathogenicity has not been reported. However, the patient’s daughter did not carry the gene mutations of *GRN* and *ErbB4* (Figs. 2 and 3).

### Characteristics, diagnosis, and treatment of the patient

The patient, a 52-year-old man, presented with a history of abnormal behavior persisting for 2 months, signaling the onset of his condition. This period marked the beginning of a gradual progression in symptoms, which included repeated shopping, taking other people’s things, constantly eating snacks, and regularly calling friends at night. The patient’s executive dysfunction involved not knowing how to cook and frequent problems when driving, including always driving off his lane. He also showed irritability, indifference and withdrawal, and other personality changes. Additionally, the patient exhibited motor symptoms such as unstable walking and consistently falling when walking. In the case of neuropsychological scales, the FTLD-modified CDR score was 4.5, while the FBI score was 47 (FBI-A score ≥ 30 indicates FTD). Brain MRI showed widening and deepening of the bilateral temporal lobe. Furthermore, brain metabolic imaging using PET-CT suggested increased PDG metabolism in the bilateral prefrontal lobe, with the abnormal energy metabolism findings signifying FTD. Moreover, gene detection results demonstrated a c.1352C > T (p.P451L) heterozygous mutation in the *GRN* gene and *ErbB4* gene has a heterozygous mutation c.256 T > C(p.Y86H). Therefore, based on the combination of the patient’s characteristics and the results of the neuropsychological scales, brain MRI and PET-CT, EMG, and gene detection, the patient was finally diagnosed with bvFTD. After establishing the diagnosis, the patient was treated with fluvoxamine maleate, lamotrigine, vitamin B6, folic acid tablets, felodipine sustained-release tablets, mecobalamin dispersible tablets, memantine, and Zaoren Anshen capsule.

In the first year of follow-up, the patient developed weakness in both lower limbs and was unable to walk independently. Bilateral Babinski signs were positive. The Amyotrophic Lateral Sclerosis Functional Rating Scale-Revised (ALSFRS-R) score was 19, indicating significant functional decline. Riluzole treatment was suggested to slow disease progression. At the 1.5-year follow-up, the patient experienced weakness in both upper limbs and was unable to hold heavy objects. Additionally, the patient developed dysarthria, dysphagia, and coughing while drinking water. The patient did not regularly take Riluzole. Two years post-diagnosis, the patient succumbed to respiratory failure.

## Discussion

FTLD is a neurodegenerative disease characterized by profound behavioral, personality, and language abnormalities [10]. Typical clinical manifestations may include social misconduct, childish behavior, eating disorders, emotional retardation, habit and ritual changes, executive dysfunction, and obvious language difficulties. These symptoms result from losing brain cells in discrete areas of the frontal lobe and/or anterior temporal lobe [11–13]. Studies have shown that social behavior is associated with the right hemisphere [14], with tissue loss in the right brain structure leading to increased social misconduct and antisocial behavior symptoms. Furthermore, apathy, disinhibition, and abnormal motor behavior significantly correlate with tissue loss in specific frontal lobe areas. Obsessive-compulsive behavior is also linked with temporal lobe atrophy [15, 16]. Interestingly, certain neurological aspects remain unaffected by FTLD. For example, several important cognitive and behavioral functions, such as memory and visual-spatial skills, are relatively intact. However, no effective drug is available to treat FTD, and the progression of the disease cannot be halted or alleviated using any current treatment [17].

Approximately 60% of the familial FTD cases are attributed to mutations in the *MAPT* and *GRN* genes and hexanucleotide amplification repeats in the open reading frame of chromosome 9 (*C9orf72*). Among them, the most common is the *C9orf72* gene mutation, accounting for about 25% of cases [18]. Other reported mutations include those of valosin containing protein (*VCP*), charged multivesicular body protein 2B (*CHMP2B***)**, TAR DNA-binding protein of 43 kDa(*TDP-43*), fused in sarcoma(*FUS*), integral membrane protein 2B [19, 20], TANK-binding kinase 1 [21], and TATA box-binding protein [22]. The gene mutation analysis of our patient showed mutations in the ALS pathogenesis-associated *GRN* gene c.1352C > T (p.P451L) and *ErbB4* gene c.256 T > C (p.Y86H). The *GRN* gene encodes a 593-amino acid (68.5 kDa) protein expressed in neurons and microglia. The *GRN* gene is located on human chromosome 17q21 and encodes granulin precursor, which is expressed in a variety of tissues, particularly epithelial cells, haematopoietic cells and neurons and microglia of the central nervous system. Granulin precursor acts as a neurotrophic factor, regulating neuronal survival and differentiation, and is also associated with stress, immune and inflammatory responses in the nervous system [23]. In Creutzfeldt–Jakob disease, motor neuron disease, and AD, *GRN* expression is increased in activated microglia. The *GRN* gene consists of 13 exons (including the non-coding exon 1) that encode 7.5 repeat sequences of granin peptide motif [24]. To date, 70 *GRN* mutations have been reported in 232 families worldwide, accounting for 5% of familial and 1% of sporadic FTD cases. All *GRN* mutations are heterozygous, resulting in the loss of 75% of the corresponding protein. The types of *GRN* mutations include frameshift, splice site, missense, nonsense, and signal peptide mutations as well as interruption in the Kozak sequence (which plays a role in translation). The mutant *GRN* mRNA is usually a prematurely terminated codon that is destroyed once detected by the cell, thereby preventing the accumulation of truncated granin domains [25]. This mechanism leads to decreased *GRN* levels in the cerebrospinal fluid, plasma, and serum, resulting in haploid deficiency. Additionally, some missense mutations may lead to non-functional or unstable *GRN* proteins [26]. *GRN*-FTD usually affects the frontal and temporal cortices, leading to behavioral changes, executive dysfunction, and language disorders. The common clinical features of *GRN*-FTD comprise abnormal behavior, language disorder, Parkinson’s syndrome, and memory disorder. The most common clinical phenotype is bvFTD, followed by nfVPPA, corticobasal syndrome, and svPPA. Furthermore, the *GRN* gene is linked with *GRN*-related FTLD with TDP-43 inclusion bodies, neuronal ceroid lipofuscinosis type 11, and primary progressive aphasia and is reported to exhibit autosomal recessive or dominant inheritance. Moreover, homozygous, compound heterozygous, and single heterozygous variants are all theoretically likely to cause disease based on the types of variation sites. The mutation site c.1C > T of the *GRN* gene has been reported as a pathogenic variant causing pathological features associated with the FTLD-TDP phenotype. The main components of the inclusion body of this variant are abnormally phosphorylated and ubiquitinated TDPs. Psychotic symptoms in *GRN* mutation carriers are mainly associated with gray matter atrophy in the anterior insular lobe, left thalamus, cerebellum, frontal lobe, parietal lobe, and occipital lobe. In our patient, abnormal behavior and executive dysfunction were the first noted symptoms. The patient gradually developed personality changes and motor symptoms during the disease course. At follow-up, the patient showed obvious mental symptoms, particularly delusional symptoms that were more common in patients with *GRN*-FTD [27]. Studies have found a significant correlation between the delusional symptoms of *GRN* mutation carriers and the left thalamus and left anterior insular lobe atrophy. Other studies have demonstrated that psychiatric symptoms in *GRN* mutation carriers were mainly related to the atrophy of the anterior insular lobe, left thalamus, cerebellum and frontal lobe, parietal lobe and occipital lobe [28, 29].

*ErbB4* is an extremely rare pathogenic gene implicated in ALS. This gene is associated with ALS type 19 and is reported to have an autosomal dominant inheritance [30]. In theory, this disease can be caused by a pathogenic mutation on one chromosome. However, according to the guidelines of the American Society of Medical Genetics and Genomics, our patient had a c.256 T > C (p.Y86H) mutation, but no such mutation has been reported at this site. Many studies have indicated that the neuromodulin 1 (*NRG1*) and *ErbB4* genes, the susceptibility genes of mental illness, are linked with the bipolar disorder and schizophrenia susceptibility genes. In the central nervous system, NRG1 activates its ErbB receptor, which affects neuronal development, migration, synaptic formation, maturation, and synaptic plasticity. When the epidermal growth factor or its homolog binds to ErbB receptors, it triggers various cellular signaling pathways, leading to multiple cellular responses that include processes from cell division to apoptosis and cell adhesion. Research has also revealed a high expression of *ErbB4* (mRNA and protein levels) in the brain tissue, which plays a key role in maintaining the working memory ability of rodents [31]. Additionally, *ErbB4* mutations have been reported and confirmed in ALS/FTD and indicated that *NRG1*-*ErbB4* activates many downstream pathway signals, including those of *PI3K*, *Akt*, and *ERK1/2*. *ErbB4* mutations also lead to a significant decrease in autophosphorylation [32]. In addition, Dols-Icardo et al. explored genetic mutations in ALS/FTD patients without the C9orf72 expansion mutation. Among the 54 investigated patients, they reported that 11 had probable pathogenic mutations, with TBK1 as the most common cause and identified possible pathogenic mutations in ERBB4, expanding our understanding of its significance in ALS/FTD co-occurrence [33]. All these results suggest that *ErbB4* gene mutation is associated with FTD. Moreover, due to recent developments in technology, gene panels, particularly Next-Generation Sequencing (NGS)-based ones, have been increasingly reported to be crucial for diagnosing genetic neuromuscular disorders quickly and cost-effectively [34], indicating the need for more translational research in this field accurate interpretation and consider advanced techniques as costs decrease, as gene panels could become increasingly used and prove to be a valuable tool for precision medicine in these patients.

In conclusion, the clinical manifestations of FTD are highly heterogeneous. Therefore, the disease is difficult to diagnose and can even be easily misdiagnosed. Currently, neuropsychological assessments, biomarkers, and gene detection tests can help establish the diagnosis of FTD. Moreover, detailed and accurate physical and neuropsychological examinations must be combined with genetic testing as soon as possible to improve patient prognosis by achieving early diagnosis and treatment. To date, *GRN* and *ErbB4* have been considered the pathogenic genes of ALS, and these two mutations have not been found in patients with ALS-FTD. Thus, collectively, this case report represents a unique instance of combined GRN and ErbB4 mutations in a patient initially presenting with bvFTD but subsequently developing motor symptoms, in line with the ALS spectrum. Our follow-up observations revealed that the patient gradually developed motor symptoms, which progressively worsened, suggesting a significant role of the ErbB4 mutation, typically associated with ALS, in the patient’s condition. This case also highlights the complexity of genetic contributions in neurodegenerative disorders and the importance of considering the full clinical trajectory in genetic analyses.

## Data Availability

The data used to support the findings of this study are available from the corresponding author upon request.
